# Two New Troglobitic Species of *Giupponia* Pérez-González & Kury, 2002 (Opiliones: Gonyleptoidea) from Caves of Bahia, Northeastern Brazil †

**DOI:** 10.3390/ani16111609

**Published:** 2026-05-25

**Authors:** Jonas E. Gallão, Maria E. Bichuette, Adriano B. Kury, Marcos R. Hara

**Affiliations:** 1 Laboratório de Estudos Subterrâneos, Departamento de Ecologia e Biologia Evolutiva, Universidade Federal de São Carlos, São Carlos 13565-905, São Paulo, Brazil; 2 Instituto Brasileiro de Estudos Subterrâneos, São Carlos 13565-545, São Paulo, Brazil; 3 Departamento de Invertebrados, Museu Nacional/UFRJ, Rio de Janeiro 20940-040, Rio de Janeiro, Brazil; 4 Escola de Artes, Ciências e Humanidades, Universidade de São Paulo (EACH USP), São Paulo 03828-000, São Paulo, Brazil

**Keywords:** neotropical region, cave fauna, karst, Pachylinae, systematics, troglomorphisms, Bahia State

## Abstract

Cave-dwelling harvestmen (Opiliones: Laniatores: Gonyleptoidea) include trogloxenes, troglophiles and troglobites, frequently represented as monotypic genera, likely reflecting taxonomic practice rather than true diversity. *Giupponia* Pérez-González & Kury, 2002 is one such case: it was erected for the blind troglobite *G. chagasi* from limestone caves in Serra do Ramalho, southwestern Bahia, Brazil, and has remained monotypic for more than two decades. Here, we describe two additional troglobitic species from caves in the same karst area, thereby expanding the genus and providing an updated diagnosis and an identification key for males. The new species share core troglomorphic traits with *G. chagasi* (complete eye loss and depigmentation) and a distinctive suite of external and genital characters that support their placement in *Giupponia*, including a theta-type dorsal scutum with wide ridged grooves, a prominent preocular mound with paired spiniform armature, an enlarged ocularial apophysis, and a characteristic penial configuration with a pyriform ventral plate, a parabolic distal cleft and a robust stylus bearing a dorsal projection, with the glans lacking dorsal/ventral processes. We further discuss the morphological evidence bearing on the suprageneric placement of *Giupponia* within Gonyleptoidea Sundevall, 1833, highlighting affinities with lineages traditionally treated in Pachylinae and possible relationships with Ampycidae Kury, 2003 or Tricommatinae Roewer, 1912. These findings underscore the hidden diversity of the Serra do Ramalho subterranean fauna and the need for integrative phylogenetic analyses to resolve the evolutionary origin of extreme troglomorphisms in Brazilian gonyleptids.

## Simple Summary

Among the most fascinating environments on Earth are caves. These places are usually humid and have little to no light, creating unique conditions for life. Throughout history, caves have often been linked to legends and mysteries, sometimes seen as homes of unknown creatures or even gateways to other worlds. Interestingly, the idea that caves shelter unusual or “strange” creatures is not entirely wrong, as new troglobitic (name given to animals that live only in caves) species are still being discovered in these environments today. In this study, we describe two new species of arachnids belonging to the order Opiliones (commonly known as harvestmen). These species belong to the genus *Giupponia*, which, until 2002, included only a single known species. These new species were found in an important region in northeastern Brazil, the Serra do Ramalho karst area, known for its large number of caves, some of them extensive and visually striking. Describing new species helps us better understand biodiversity and provides important information to support the conservation of these unique environments and their fauna.

## 1. Introduction

Opiliones Sundevall, 1833 is one of the most species-rich orders of arachnids, with nearly 6900 valid species [1], surpassed only by Araneae Clerck, 1757 (spiders) and “Acari” (ticks and mites), and distributed on all continents except Antarctica [2]. Opiliones inhabit all ecosystems and are often found on vegetation, on the ground, under rocks, tree trunks, and stone walls [2]. Some species have also successfully colonized subterranean habitats, such as caves, mainly due to their nocturnal habits, omnivory, and tolerance to high relative humidity. There are representatives of Opiliones in the three main ecological-evolutionary categories of cave dwellers [3,4]: trogloxenes, troglophiles, and troglobites. Trogloxenes are species whose epigean (i.e., outside caves) populations use subterranean resources and necessarily have to leave the cave periodically, such as several species of Goniosomatinae Mello-Leitão, 1935, especially in the genera *Serracutisoma* Roewer, 1930 and *Goniosoma* Perty, 1833. Troglophiles are species whose populations inhabit both epigean and subterranean habitats, e.g., several species of *Eusarcus* Perty, 1833. Finally, troglobites are those that are restricted to and obligate cave-dwellers, e.g., all species of *Iandumoema* Pinto-da-Rocha, 1997.

Recently [5,6,7,8,9], Brazilian cave-dwelling harvestmen have received renewed interest, accompanied by the arrival of new researchers in the field and also promoted by Brazilian environmental laws [8]. Among these Brazilian cave-associated species, most belong to the Neotropical family Gonyleptidae Sundevall, 1833, the second most species-rich family of the suborder Laniatores [1].

Historically, newly described gonyleptid troglobitic species have often been classified into new genera, remaining as monotypic groupings: that is the case of *Pachylospeleus* Šilhavý, 1974a for *Pachylospeleus strinatii* Šilhavý, 1974a from caves in the state of São Paulo and *Giupponia* Pérez-González & Kury 2002 for *G. chagasi* Pérez-González & Kury, 2002 from caves in the state of Bahia. A possible explanation for this taxonomical decision might be due to the numerous modifications that those species undergo related to the cave evolution of these lineages, blurring their phylogenetic relationships. The only exception so far is *Iandumoema* Pinto-da-Rocha, 1997, originally proposed for *I. uai* [10] from a cave in the state of Minas Gerais that nowadays harbors five additional species: (*I. setimapocu* Hara & Pinto-da-Rocha, 2008, *I. smeagol* Pinto-da-Rocha et al., 2015, *I. cuca* Ázara et al., 2020, *I. gollum* Ázara et al., 2020, and *I. stygia* Ázara et al., 2020). The few troglobitic gonyleptid species that were not classified in a new genus belong to *Discocyrtus* Holmberg, 1878 and *Eusarcus*, both known as speciose genera: *Discocyrtus pedrosoi* Kury, 2008 and *Eusarcus elinae* Kury, 2008, respectively. All troglobitic species mentioned so far belong to the polyphyletic subfamily Pachylinae Sørensen, 1884.

After more than twenty years, we finally removed *Giupponia* from its monotypic status, with the description of two new species from caves in the Serra do Ramalho karst area, in the same region as its type species. We also propose putative synapomorphies for the genus and discuss its phylogenetic relationships based on morphological grounds.

## 2. Materials and Methods

The depository of the material is the Laboratório de Estudos Subterrâneos, Universidade Federal de São Carlos, São Carlos (LES/UFSCAR; curator: Maria E. Bichuette). The material is preserved in 70% ethanol. We illustrated the general external morphology of the specimens immersed in 70% ethanol using a Leica M80 stereomicroscope (Leica/ Heerbrugg, Switzerland) with a camera lucida (piece number 10446193) (mainly appendages and body). We dissected the male genitalia and clarified them with glycerin following [11,12]. We drew the male genitalia using an Olympus BX41 optical compound microscope (Olympus/ Tokyo, Japan) with a camera lucida (piece number U-DA 9E12245). Photographs 6B and 6C of *Giupponia lemmyi* sp. nov. were taken with a Leica DFC 295 camera (Leica/ Heerbrugg, Switzerland) attached to a Leica M205C stereomicroscope (Leica/ Heerbrugg, Switzerland) with a Planapo 1.0× objective. Figures were produced from stacks of images on Leica Application Suite (LAS) software v3.7 (Leica/ Wetzlar, Germany). All the measurements are in millimeters. Coloration refers to specimens preserved in 70% ethanol. The nomenclature of structures and topology follows [12] with minor modifications to accommodate the taxa studied. The nomenclature of the macrosetae of the penis and the shape of the dorsal scutum follow [13,14], respectively. We used the following abbreviations: CL, carapace length; Co, coxa; CW, carapace width; DS, dorsal scutum; DSL, dorsal scutum maximum length; DSW, dorsal scutum maximum width; Fe, femur; Me, metatarsus; MSA–D, macrosetae A–D of the penis ventral plate; Pa, patella; Ta, tarsus; and Ti, tibia. We only included the characters that differ from the male in the female descriptions.

A formal differential diagnosis is not provided because *Giupponia* cannot presently be contrasted with a restricted set of clearly comparable genera. The relevant characters are not individually exclusive to the genus, although their combination is distinctive. A broad contrast at the superfamilial level would require extensive comparisons with numerous unrelated taxa sharing isolated character states, resulting in a cumbersome and poorly informative diagnosis.

### Study Area

**Figure 1:**
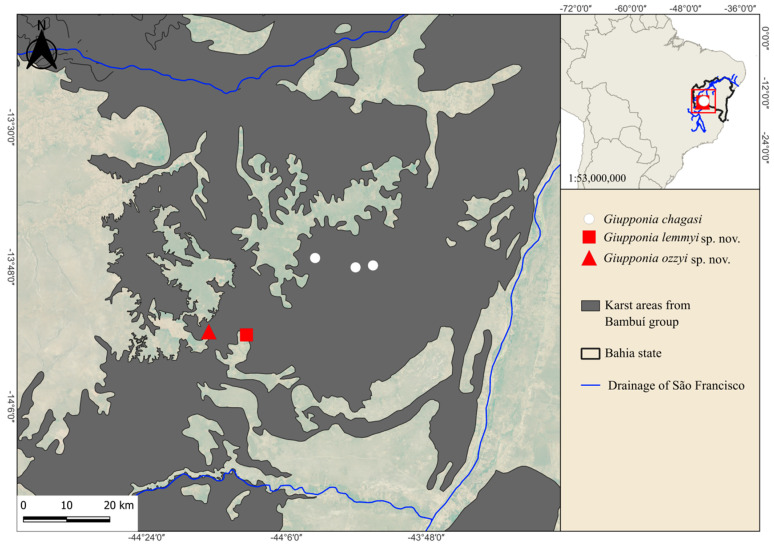
Known distribution of *Giupponia* spp., highlighting the karst formations. Map confection: Marcus VdSA Duarte.

**Figure 2:**
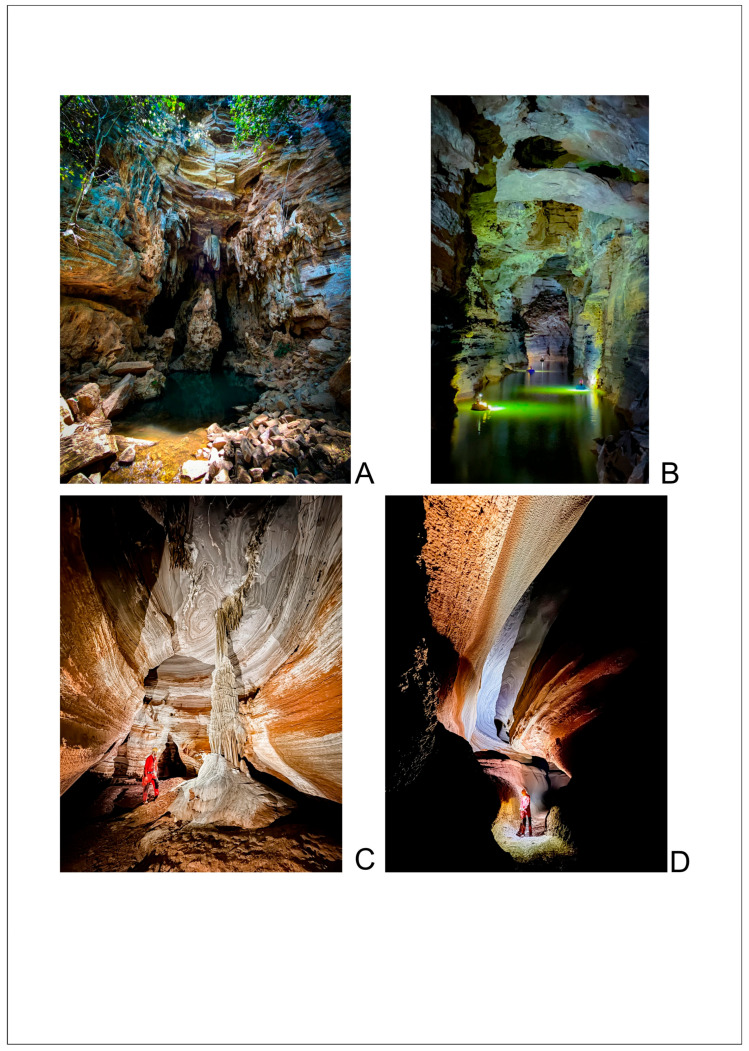
Habitats of *Giupponia* spp. herein described. (**A**), entrance of cave Gruna Boca da Lapa; (**B**), same, interior. (**C**), entrance of cave Gruna do Engrunado, (**D**), same, interior. Both caves are in the municipality of Feira da Mata.

The Serra do Ramalho karst area, in the southwestern State of Bahia, lies within the Bambuí geomorphological group (Figure 1). The Serra do Ramalho karst area holds a high number of caves, mostly without legal protection, with some of them exceeding 15 km of extension [15]. The Serra do Ramalho karst area comprises the municipalities of Coribe, Feira da Mata, Carinhanha, and Serra do Ramalho, with the shape of several plateaus composed of carbonate rocks (limestone) of the Bambuí geomorphological group. Moreover, carbonate rocks crop out in the region, forming plateaus parallel to the São Francisco River [15]. According to Köppen criteria, despite the semiarid surroundings (BSh) at east, the region has a tropical dry climate spot (“Aw”), with a slightly different dry climate spot (Aw’) at west, with annual precipitation of approximately 640 mm and subterranean waters mainly exploited for human consumption [15,16]. The surrounding vegetation consists of Caatinga, composed of mesophytic and xeromorphic forests interspersed with Cerrado (Brazilian savannah-like vegetation). The caves inhabited by the species described here are from Gruna Boca da Lapa and Gruna do Engrunado (Figure 2), both in the Municipality of Feira da Mata.

## 3. Results

### Taxonomy

Genus *Giupponia* Pérez-González & Kury, 2002

*Giupponia* [17]: 44; [6]: 77 (cat).

**Figure 3:**
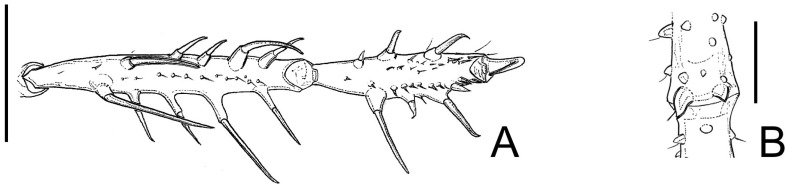
*Giupponia chagasi*. Male (LES 0031761): (**A**), right pedipalpal tibia–tarsus, ventral view; (**B**), right femur IV apex, dorsal view.

Type species: *Giupponia chagasi* Pérez-González & Kury, 2002; Figure 3

Diagnosis

**Figure 4:**
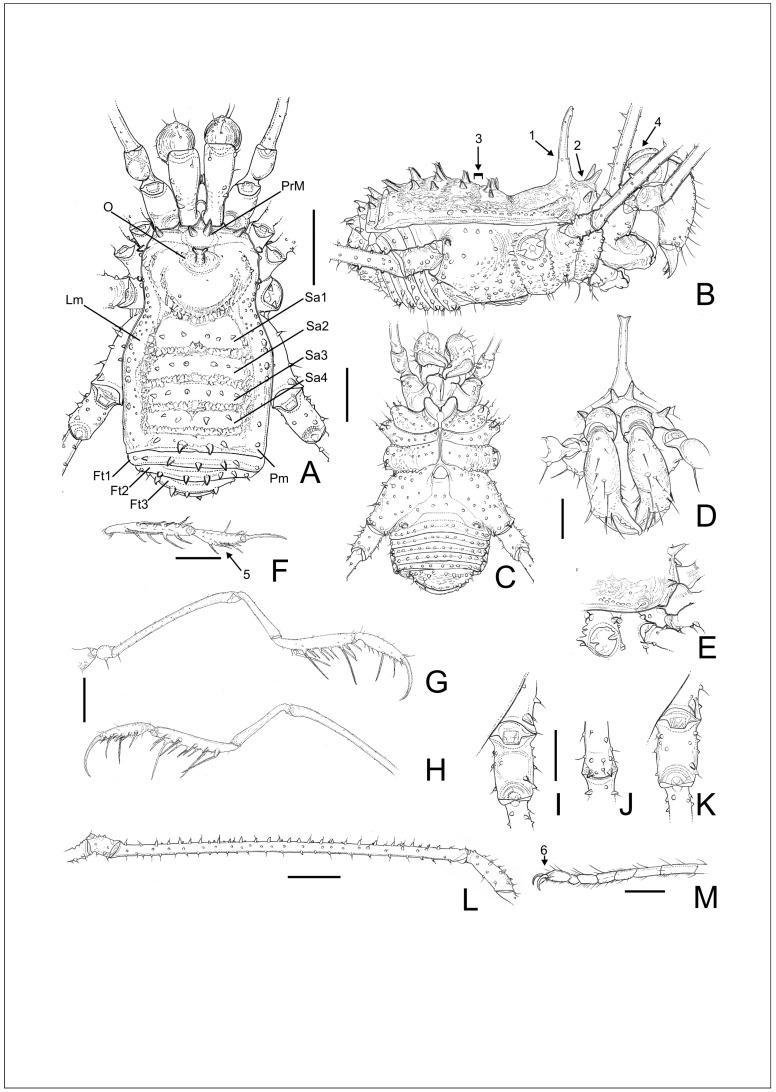
*Giupponia lemmyi* sp. nov. Male (holotype): (**A**), habitus, dorsal view; (**B**), same, right lateral view; (**C**), same, ventral view (averaged size tubercles omitted); (**D**), ocularium, anterior view; (**E**), dorso-right lateral view of ozopore area; (**F**), right pedipalpal tibia–tarsus, ventral view; (**G**), right pedipalp, ectal view; (**H**), same, mesal view; (**I**), male right trochanter IV, dorsal view; (**J**), right femur IV apex, dorsal view; (**L**), right coxa–patella IV, retro-lateral view; (**M**), right tarsus IV, pro-lateral view. (**K**), Female paratype right trochanter IV, dorsal view. Abbreviations: Ft1–3, free tergites I–III; Lm, lateral margin of dorsal scutum; O, ocularium; Pm, posterior margin of dorsal scutum; PrM, preocular mound; Sa1–4, scutal areas I–IV. Numbered arrows indicate diagnostic characters of the genus: 1, ocularium without eyes bearing long apophysis; 2, armed Prm; 3, large scutal groove (transversal bracket indicates its length); 4, cheliceral segment I leaning dorsad; 5, asymmetric pedipalpal tarsus; 6, absence of tarsal process. (**A**,**B**), at same scale; (**D**,**E**), at same scale; (**F**–**H**) at same scale; (**I**–**K**) at same scale. Scale bars: (**A**–**C**,**F**–**H**,**L**): 1.0 mm; (**D**,**E**,**I**–**K**,**M**): 0.5 mm.

**Figure 5:**
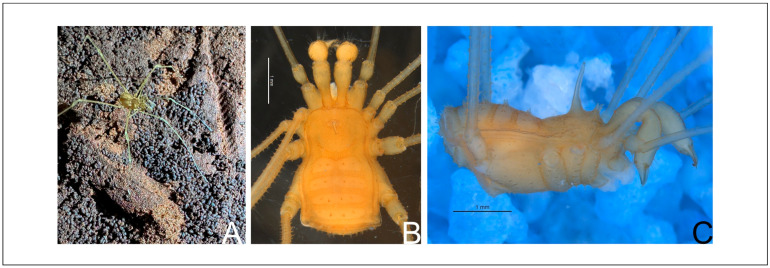
*Giupponia lemmyi* sp. nov. Female (paratype). (**A**), living specimen in its natural habitat; (**B**), habitus, dorsal view; (**C**), same, right lateral view.

**Figure 6:**
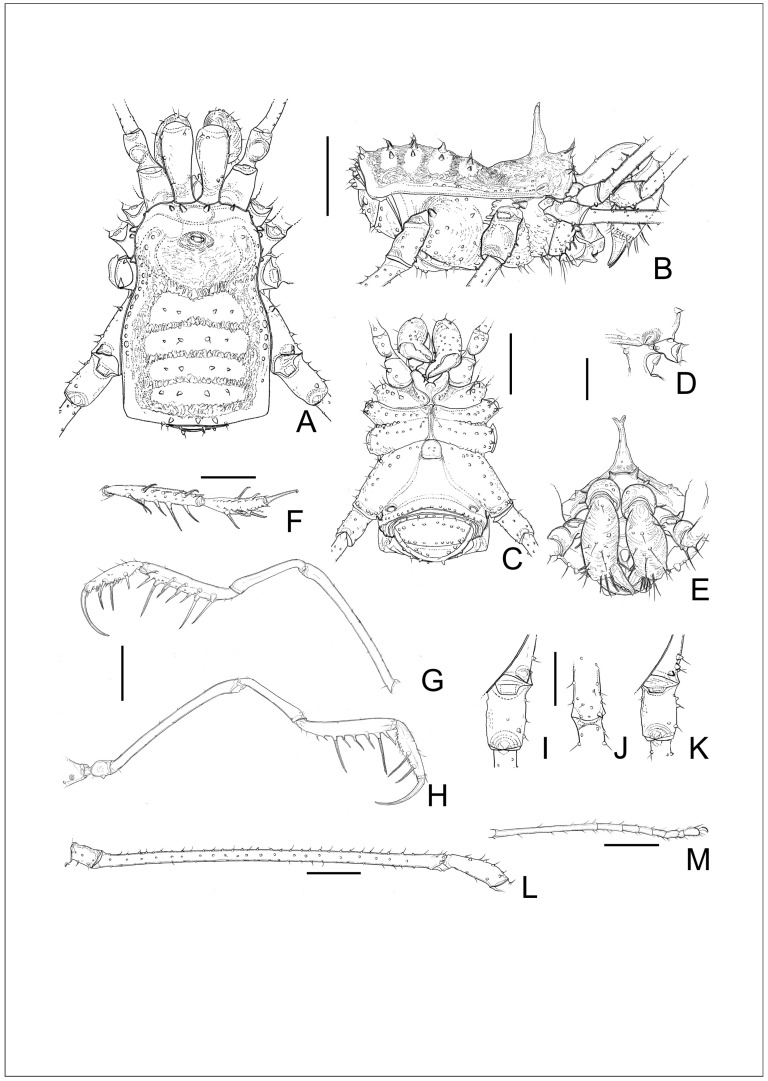
*Giupponia ozzyi* sp. nov. Male (holotype): (**A**), habitus, dorsal view; (**B**), same, right lateral view; (**C**), same, ventral view (averaged size tubercles omitted); (**D**), dorso-right lateral view of ozopore area; (**E**), ocularium, anterior view; (**F**), right pedipalpal tibia–tarsus, ventral view; (**G**), right pedipalp, mesal view; (**H**), same, ectal view; (**I**), right trochanter IV, dorsal view; (**J**), right femur IV apex, dorsal view; (**L**), right coxa–patella IV, retro-lateral view; (**M**), right tarsus IV, retro-lateral view. (**K**), Female paratype right trochanter IV, dorsal view. (**A**,**B**), at same scale; (**D**,**E**), at same scale; (**F**–**H**) at same scale; (**I**–**K**) at same scale. Scale bars: (**A**–**C**,**F**–**H**,**L**): 1.0 mm; (**D**,**E**,**I**–**K**,**M**): 0.5 mm.

**Figure 7:**
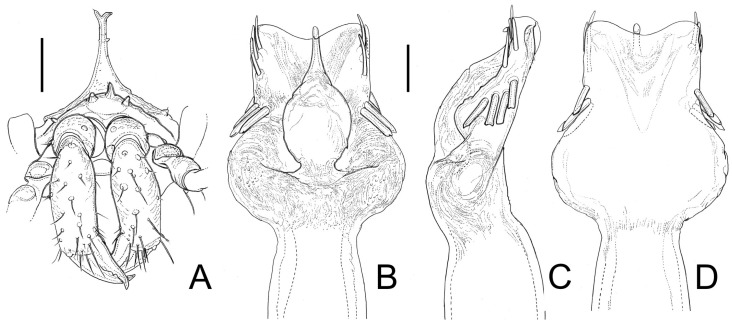
*Giupponia ozzyi* sp. nov. Male (paratype): (**A**), ocularium, anterior view; (**B**), distal penis, dorsal view; (**C**), same, right lateral view; (**D**), same, ventral view.

**Figure 8:**
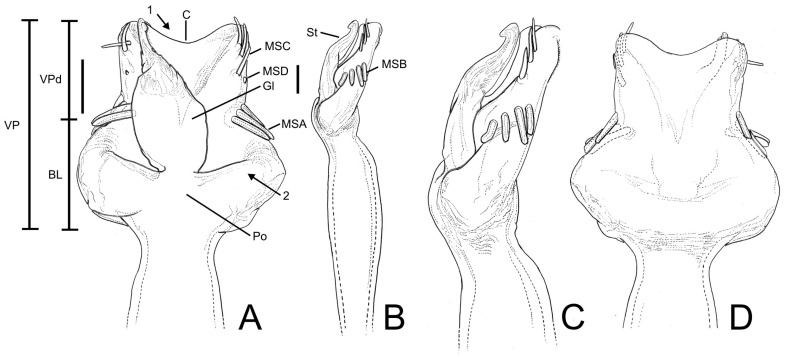
*Giupponia lemmyi* sp. nov. Male (holotype), penis: (**A**), dorsal view; (**B**), right lateral view, panoramic view; (**C**), same, close up; (**D**), ventral view. Abbreviations: BL, basal lobes of VP; C, cleft on VP anterior margin; Gl, glans sac; Po, podium; St, stylus of the glans; VP, ventral plate of penis; VPd, ventral plate distal part; MSA–D, macrosetae A–D of the VP. Numbered arrows indicate diagnostic characters of the genus: 1, parabolic cleft on VP anterior margin; 2, podium largely fused with basal lobes (BL) of VP.

Gonyleptoidea without eyes (Figure 4A,B, Figure 5B,C and Figure 6A,B), ocularium with an unbranched or bifid long spiniform apophysis (Figure 4B,D, Figure 5C, Figure 6B and Figure 7A). Dorsal scutum (DS) type theta (Figure 4A, Figure 5B and Figure 6A), with four scutal areas, free tergites and sternites unarmed or each with central large pointed tubercles. Scutal area I undivided, scutal grooves wide and ridged (Figure 4A,B, Figure 5B,C and Figure 6A,B). Anterior margin of DS with preocular mound bearing two paramedian spiniform apophysis, plus one on each side (Figure 4A, Figure 5A and Figure 6A). Cheliceral segment I leaning dorsad, with weakly marked bulla. Pedipalpal tarsus asymmetric, mesal face swollen (Figure 3A, Figure 4F and Figure 6F) with two mesal ventral rows of small setae. Pedipalpal tibia with an internal ventral row of small setae (Figure 3A, Figure 4F and Figure 6F). Coxa IV reaching scutal area III (Figure 4A, Figure 5B and Figure 6A), males with unbranched or trifid small pro-dorsal apical apophysis and a small, unbranched retro-apical one. Femur IV without remarkable large apophysis or spines on its length (Figure 4L and Figure 6L). Tarsus III–IV without tarsal process. Ventral plate with parabolic cleft, three pairs of thick, blunt or truncated MS A, one pair of thick, short, ventrally placed MS B, and three pairs of cylindrical pointed MS C (Figure 7B–D and Figure 8). Glans without dorsal or ventral processes, podium partially fused to ventral plate (Figure 7B,C and Figure 8A,B). Stylus apex bearing a dorsal projection. Relative length of legs: II-IV-III-I. Tarsal segmentation male 8–11(3)/23–37(4–5)/7/7–8, female 7–10(3)/27–36(3–5)/7/7–8. Calcaneus I more than twice as long as astragalus, calcanei II-III a little shorter, calcaneus IV much shorter than astragalus.

Included species

*Giupponia chagasi* Pérez-González & Kury, 2002 (type species), *Giupponia lemmyi* sp. nov., and *Giupponia ozzyi* sp. nov.

Geographical distribution

Brazil: Bahia state: caves in the municipalities of Carinhanha and Feira da Mata (Figure 1).

Key to males of *Giupponia*

1. Ocularium with an unbranched, long spiniform apophysis; scutal areas I–IV unarmed (i.e., without large tubercles compared to other covering the same area); and coxa IV with trifid pro-dorsal apical apophysis                                 *G. chagasi*.

Ocularium with a bifid, long spiniform apophysis (Figure 4A,D and Figure 6A,E); scutal areas I–IV with paired armature (i.e., with a pair of large tubercles conspicuously standing out among the others covering the same area) (Figure 4A,B and Figure 6A,B); and coxa IV with unbranched pro-dorsal apical apophysis (Figure 4I and Figure 6I).

2. Spiniform apophysis higher than the ocularium (in lateral view) (Figure 4B); femur IV with retro-dorsal and retro-lateral rows of pointed and conspicuously high tubercles (Figure 4L); and patella IV with retro-dorsal row of pointed and conspicuously high tubercles                                                       *G. lemmyi* sp. nov.

Spiniform apophysis as high as the ocularium (in lateral view) (Figure 6B); femur IV with retro-dorsal and retro-lateral rows of similar-sized, slightly conical and blunt tubercles (Figure 6L); and patella IV with retro-dorsal row of slightly pointed tubercles (as high as the other covering the podomere)                               *G. ozzyi* sp. nov.

*Giupponia chagasi* Pérez-González & Kury, 2002

(Figure 3A,B)

*Giupponia chagasi* [17]: 44, figs 1–12; [18]: 50 (cit), 57 (cit); [19]: 259 (cit); [20]: 507 (cat); [NO_PRINTED_FORM] [21]: 1816 (cit); [6]: 5 (cit), 77 (cat); [4]: 8 (cit.); [22]: 27–29 (cit); [23]: 11 (cit).

Material examined

BRAZIL. Bahia: Carinhanha (Lapa do Boqueirão), 26.iv.2022, Bichuette, Gallão & Horta leg., 2 ma (LES 031761); idem (Gruna Pedro Cassiano), 10.ix.2021, Bichuette & Gallão leg., 4 fe (LES 027769).

Note: Male type material destroyed in the fire at Museu Nacional do Rio de Janeiro (MNRJ) [24]. Most of the paratypes in other depositories are females, which would not help to update the diagnosis here proposed, as it is for males. In gonyleptids, usually the male bears the diagnostic features of the species while females do not, often being identical, especially between phylogenetically close species.

Diagnosis for males

*Giupponia chagasi* can be distinguished from the remaining species of the genus by the unbranched, high spiniform apophysis on the ocularium, unarmed scutal areas, and the trifid pro-dorsal apical apophysis on coxa IV.

Note: As other species of the genus, *G. chagasi* exhibits the swollen mesal face of the pedipalpal tarsus (Figure 3A), albeit in a slightly attenuated form, and two ventral internal rows of small setae. We also noted that (i) both males and females exhibit a small, unbranched retro-apical apophysis on coxa IV, and (ii) males bear a pair of pointed tubercles on the dorso-apical face (the retro-dorsal one being the largest) of the femur IV (Figure 3B). Pérez-González and Kury (2002) did not mention it, but the lateral view of the habitus in *G. chagasi*’s description (Figure 3 in Peréz-González & Kury 2002) depicts the ridged surface on the scutal grooves.

*Giupponia lemmyi* sp. nov.

(Figure 4, Figure 5 and Figure 8)

Zoobank: http://zoobank.org/urn:lsid:zoobank.org:act:9B57B47A-7473-4684-BDFD-7DD1195A11FA, accessed on 20 May 2026.

Etymology: the species honors Lemmy Kilmister, English musician and iconic heavy metal and hard rock singer (and songwriter too), and founder of the rock band Motörhead.

Type data

Holotype. BRAZIL. Bahia: Feira da Mata (Gruna Boca da Lapa), 15.x.2020, Bichuette, Gallo, Horta & Gallão leg. (LES 027765).

Paratypes. BRAZIL. Bahia: Feira da Mata (Gruna Boca da Lapa), 15.x.2020, Bichuette, Gallo, Horta & Gallão leg., 1 ma & 1 fe (LES 0031759).

Diagnosis

*Giupponia lemmyi* sp. nov. can be distinguished from other species of the genus by the combination of the following characters: ocularium with a bifid high-spiniform apophysis, this higher than the ocularium (in lateral view); all scutal areas with a paramedian pair of large spiniform apophyses; coxa I with reduced or without a pro-dorsal apophysis; coxa IV with a small pro-dorsal apical spiniform apophysis; and femur IV with retro-dorsal and retro-lateral rows of conspicuously large, pointed tubercles.

Description

Male holotype (LES 027765)

Dorsum (Figure 4A,B,D,E). Measurements of the body parts and legs are in Table 1 and Table 2. Dorsal scutum (DS) outline type theta (θ). Scutal areas with pointed tubercles. Anterior margin of DS with conspicuous preocular mound bearing a pair of spiniform apophysis plus a pointed tubercle on each side. Ocularium slightly placed anteriad, with sparsely distributed small tubercles and a bifid, high spiniform apophysis (higher than the ocularium’s). Carapace rugged, with a large, pointed tubercle between ocularium and lateral margin of DS on each side, plus a few tubercles. All scutal areas, posterior margin of DS, and free tergites I–III, each with a transversal row of pointed tubercles. All scutal areas, posterior margin of DS, and free tergite II, each with a paramedian pair of large tubercles. Scutal area I undivided. Free tergites I and III, each with a large central spiniform tubercle. Scutal grooves wide and ridged. Lateral margin of DS with a regular external row of tubercles increasing in size posteriorly up to scutal area III and an irregular internal row of small tubercles. Anal operculum tuberculate.

Venter (Figure 4C). Coxae I–IV with distal large tubercles; coxae I–II with a longitudinal central row of large tubercles. Coxae I–II with similar apical width. Coxae III–IV tuberculate, with similar apical width, each roughly 1.5 times that of coxa I. Coxa IV retro-apical margin close to but not in contact with the posterior margin of the genital sternite. Posterior margin of genital sternite and free sternites each with a row of tubercles.

Chelicerae (Figure 4A,B,D): Segment I elongate (as long as the carapace), leaning dorsad, with weakly marked bulla, four ectal and one mesal tubercles. Segment II, fixed finger, and segment III, movable finger toothed.

Pedipalps (Figure 4F–H): Coxa dorsal face basally inflated, with three ventro-ectal large setiferous tubercles (apical largest). Trochanter dorsal face smooth, inflated; ventral face with two–three setiferous tubercles (mesal largest). Femur dorsal face unarmed, with sparsely distributed tubercles; mesal face unarmed; ventral face with one large basal setiferous tubercle and five–six along its length. Patella unarmed. Tibial setation: mesal IiiIi, ectal IiiIi/IiiiIi. Tarsal setation: mesal IiiIi/IiiiIi, ectal II.

Legs (Figure 4I,J,L,M): Coxa I with a reduced pro-dorsal apophysis and a conspicuous retro-dorsal one; coxae II–III each with a pro- and a retro-dorsal apophysis; the apex of the retro-dorsal apophysis of coxa II fused with the pro-dorsal one of coxa III. Coxa IV reaching as far as the scutal area III, with few pointed tubercles on the dorsolateral face, a slightly large, pointed pro-dorsal apical tubercle and a retro-dorsal apical one; this fused to the posterior margin of the genital sternite. Trochanters I–IV tuberculate and unarmed. Trochanter IV with slightly large tubercles on the pro-lateral, retro-lateral and pro-dorsal subapical faces. Femora I–III unarmed, with one pair of slightly large, pointed tubercles on the dorso-apical face. Femur IV with a retro-dorsal row of conspicuously large, pointed tubercles; the dorso-apical face unarmed with a pair of slightly large, pointed tubercles. Femur IV with a retro-lateral row of spaced, large, pointed tubercles; ventro-apical face unarmed. Patellae I–IV unarmed; patella IV with pointed tubercles sparsely distributed. Tibiae and metatarsi I–IV unarmed. Tarsal counts: 9–10 (3); 31 (4); 7; 8.

Penis (Figure 8): Ventral plate (VP) pyriform, with parabolic cleft on anterior margin, and “V” shaped depression where the glans’ stylus rests. VP with three pairs of cylindrical blunt or slightly pointed MS A, one pair of ventrally placed MS B (similar size as MS A), three pairs of slender and pointed MS C, and one pair of short MS D (close to the basalmost MS C). VP swollen close to the pairs of MS A. Podium apically widened, largely fused with the basal lobes of VP. Glans sac columnar, without dorsal or ventral processes. Stylus thick, with apex dorsally projected.

Coloration. Pale brown, whiter shade of pale on the thinner portion of the appendages (metatarsi, tarsi).

Female paratype (LES 0031759)

Dorsum (Figure 5A–C). Measurements of the body parts and legs are in Table 3 and Table 4. Anterior margin of DS with a conspicuous preocular mound bearing three spiniform apophysis. Carapace with a large, pointed tubercle between the ocularium and the lateral margin of DS on each side. Posterior margin of DS and free tergite II each with a paramedian pair of slightly large, pointed tubercles. Free tergites I and III, each with tubercles increasing in size to the middle. Coxae I–IV idem male, but coxa I with pro- and retro-dorsal apophyses. Coxa IV reaching as far as the scutal area III, with tubercles slightly smaller than those of the male (Figure 4K). Femur IV tubercles smaller than those of males, except for those on the retro-dorsal row of large, pointed tubercles. Tarsal counts: 9–10 (3)/28–30 (4)/7/8.

Variation in males (N = 2): Measurements: See Table 1 and Table 2. Pedipalps: tibial setation: ectal IiiIi/IiiiIi/IIiiiIi. Free tergite III with or without a pair of large paramedian tubercles. Tarsal counts: 9–11(3)/30–31(4–5)/8/7–8.

*Giupponia ozzyi* sp. nov.

(Figure 6 and Figure 7)

Zoobank: http://zoobank.org/urn:lsid:zoobank.org:act:7387E545-3D33-4B5E-B58F-83260BA255BA, accessed on 20 May 2026.

Etymology: the species honors Ozzy Osbourne, another iconic heavy metal and hard rock singer (and songwriter too) who co-founded the band Black Sabbath. On an additional note, he is also known for biting a bat (a cave-related animal) during a concert.

Type data

Holotype: BRAZIL. Bahia: Feira da Mata (Gruna do Engrunado), 08.vii.2021, Bichuette, Gallo, Horta & Gallão leg. (LES 027766).

Paratypes: BRAZIL. Bahia: Feira da Mata (Gruna do Engrunado), 08.vii.2021, Bichuette, Gallo, Horta & Gallão leg., 1 ma and 1 fe (LES 0031760).

Diagnosis

*Giupponia ozzyi* sp. nov. can be distinguished from other species of the genus by the combination of the following characters: ocularium with a bifid high-spiniform apophysis, this being as high as the ocularium (in lateral view); all scutal areas each with a paramedian pair of spiniform apophyses; coxa I with reduced or without pro-dorsal apophysis; coxa IV with a small pro-dorsal apical spiniform apophysis; femur IV without a row of large, conspicuous tubercles.

Description

Male holotype (LES 027766)

Dorsum (Figure 6A,B,D,E): Measurements of the body parts and legs are in Table 5 and Table 6. Dorsal scutum (DS) outline type theta (θ). Scutal areas with pointed tubercles. Anterior margin of DS with conspicuous preocular mound bearing a pair of spiniform apophyses plus a pointed tubercle on each side. Ocularium slightly placed anteriad, with sparsely distributed small tubercles and a bifid, high-spiniform apophysis (as high as the ocularium). Carapace rugged, with a large, pointed tubercle between ocularium and lateral margin of DS on each side, plus a few tubercles. All scutal areas, posterior margin of DS, and free tergites I–III, each with a transversal row of pointed tubercles. All scutal areas and free tergites I and III each with a paramedian pair of large tubercles. Scutal area I is undivided. Posterior margin of DS with three large, pointed central tubercles, and free tergite II with a large central spiniform tubercle. Scutal grooves wide and ridged. Lateral margin of DS with a regular external row of tubercles increasing in size posteriorly up to scutal area III and an irregular internal row of small tubercles. Anal operculum tuberculate.

Venter (Figure 6C): Coxae I–IV with distal large tubercles; coxae I with a longitudinal central row of large tubercles. Coxae I–IV tuberculate, coxae I–II with similar apical width. Coxae III–IV with similar apical width, each roughly 1.5 times that of coxa I. Coxa IV retro-apical margin close to but not in contact with the posterior margin of the genital sternite. Posterior margin of genital sternite and free sternites each with a row of tubercles.

Chelicerae (Figure 6A,B,E). Segment I elongate (as long as carapace), leaning dorsad, with weakly marked bulla, 3–4 ectal and 1–2 mesal tubercles. Segment II, fixed finger, and segment III, movable finger toothed.

Pedipalps (Figure 6F–H): Coxa dorsal face basally inflated and two–three ventro-ectal large setiferous tubercles (apical/subapical largest). Trochanter dorsal face smooth, inflated; ventral face with three setiferous tubercles (mesal sub-apical largest). Femur dorsal face unarmed, with sparsely distributed tubercles; mesal face unarmed; ventral face with one large basal setiferous tubercle and seven along its length. Patella unarmed. Tibial setation: mesal IiiIi, ectal IiiiIi. Tarsal setation: mesal IiIi, ectal II.

Legs (Figure 6I,J,L,M): Coxae I–III, each with a pro-dorsal and a retro-dorsal apophysis; the apex of the retro-dorsal apophysis of coxa II, fused with the pro-dorsal one of coxa III. Coxa IV reaching as far as the scutal area III, with few pointed tubercles on the dorsolateral face, a slightly large, pointed pro-dorsal apical tubercle, and a comparable retro-dorsal apical one, this not fused to the posterior margin of the genital sternite. Trochanters I–IV tuberculate and unarmed. Trochanter IV with slightly large tubercles on the pro-lateral, retro-lateral and pro-dorsal subapical faces. Femora I–IV unarmed, with one pair of slightly large tubercles on the dorso-apical face. Femur IV with rows of similarly sized pointed tubercles; ventro-apical face unarmed. Patellae I–IV unarmed; patella IV with pointed tubercles sparsely distributed. Tibiae and metatarsi I–IV unarmed. Tibiae III–IV with slightly swollen pro-lateral subdistal area. Tarsi III–IV without tarsal process. Tarsal counts: 8–9 (3)/28(4)/7/8.

Penis (Figure 7B–D): VP pyriform, with parabolic cleft on anterior margin and “V” shaped depression where the glans’ stylus rests. VP with three pairs of cylindrical, blunt or slightly bicuspid MS A, one pair of ventrally placed MS B (similar size as MS A) and close to MS C3, three pairs of slender and pointed MS C, without MS D. VP swollen close to the pairs of MS A. Podium apically widened, largely fused with basal lobes of VP. Glans sac columnar, without dorsal or ventral processes. Stylus thick, with apex dorsally projected.

Coloration: Pale brown, whiter shade of pale on the thinner portion of the appendages (metatarsi, tarsi).

Female paratype (LES 0031760)

Dorsum: Measurements of the body parts and legs are in Table 7 and Table 8. Posterior margin of DS and free tergite II each with a paramedian pair of slightly large, pointed tubercles. Free tergite I with three large, pointed central tubercles. Free tergite II with a paramedian pair of large tubercles. Pedipalpal coxa with two ventro-ectal large setiferous tubercles (the apical being the largest). Pedipalpal tarsal setation: mesal IiIi(i)/iiIi(i). Coxa IV reaching as far as the scutal area III, with tubercles slightly smaller than those of the male (Figure 6K). Tarsal counts: 7 (3)/27–28 (3)/7/7–8.

Variation in males (N = 2): Measurements: See Table 5 and Table 6. Anterior margin of DS with conspicuous preocular mound bearing two (a pair) or three spiniform apophyses (Figure 6E and Figure 7A). Posterior margin of DS with two (paramedian pair) or three central large, pointed central tubercles. Chelicerae segment I with or without ectal central dorsal large tubercle. Tarsal counts: 8–9(3)/23–28(4)/7/8.

## 4. Discussion

### 4.1. Taxonomy

At the time of its description, ref. [17] placed *Giupponia* in Pachylinae (Gonyleptidae), where it remains to this day. However, the internal phylogenetic relationships of Pachylinae (and Gonyleptidae) have changed quite drastically over the past two decades, due to the increasing availability of morphological and molecular data [25,26,27,28,29]. The composition of Pachylinae began to change as new subfamilies were proposed (e.g., Ampycinae Kury, 2003, now Ampycidae) and older ones received new circumscriptions, notably Gonyassamiinae Soares & Soares, 1988 [30,31] and Tricommatinae Roewer, 1912 [26]. The polyphyletic nature of the Pachylinae was later corroborated by molecular phylogenies [29,32], revealing Pachylinae sensu stricto, a clade mainly composed of Chilean and Argentinean species together with at least six other main lineages, most of them composed of Brazilian species. Further studies, many of them based on morphological analyses, continued to dismantle Pachylinae, leading to the recognition of new groups such as Roeweriinae [27], Askawachidae [33] and Neopachylinae [34]. Despite these arrangements, *Giupponia* has remained untouched in Pachylinae, and its current placement deserves further consideration.

We agree with [17] that male genitalia provide the most reliable characters for assessing subfamilial placement. According to these authors, *Giupponia* should be placed in Pachylinae, since many species then assigned to this subfamily possess a naked stylus (without dorsal or ventral process of the glans) and a subrectangular ventral plate. However, as stated above, several changes in the subfamily have taken place, and *Giupponia*’s rather unique external body and appendages, together with its troglomorphic features, might have prevented its transfer from Pachylinae. Since *Giupponia* lacks the ventral process of the glans, we can rule out suprageneric groups that possess it: Pachylinae sensu stricto, Gonyassamiinae, Neopachylinae, and Askawachidae. Those groups are also ruled out based on the stylus features of the glans as follows: Pachylinae sensu stricto have a slender, slightly sigmoid stylus that points dorsally; Gonyassamiinae have a stylus swollen dorso-ventrally [31]; Neopachylinae have an apical stylus with spines [27]; and finally, Askawachidae have a carunculus in the stylus [33].

Among the other suprageneric groups lacking a ventral process, Roeweriinae, Ampycidae and Tricommatinae remain. In Roeweriinae, the glans stylus is typically sigmoid, strongly bent dorsad proximally and somewhat curved ventrad distally, with the apex bearing winglets. The ventral plate shape of Roeweriinae resembles a roughly apical-basally symmetric hexagon, with the widths of the distal and basal margins approximately one-third of the basal lobe width (except in Bunopachylus Roewer, 1943, where the distal margin is 1.5 to 2 times the basal width) in situ, and with the basal half depressed dorso-ventrally. Furthermore, in Roeweriinae, MS A–B are located on the basal lobe. None of those characters matches those of *Giupponia*, which possesses a robust stylus with a dorsal projection at its apex that rests on a V-shaped depression of the ventral plate. The ventral plate of *Giupponia* is pear-shaped, but MS A–B are displaced from the basal lobe, lying just posterior to the distal part of the ventral plate. Finally, the ventral plate of *Giupponia* bears dorso-lateral elevations on the basal lobe that embrace the basal half of the glans sac.

The male genital characters of *Giupponia* appear to be quite close to those of Ampycidae [30,35,36,37], although the ventral plate shape and the placement of MS A–B vary among the species. In addition to male genital characters, it is noteworthy that an undivided scutal area I can also be seen in some Ampycidae, such as species of *Glysterus* Roewer, 1931, and *Hernandarioides plana* Pickard-Cambridge, 1905 (unpubl. data), as well as in the loss of conspicuous sexual dimorphism (*Ampycella spiniventris* Roewer, 1929). Furthermore, the wide scutal grooves, a rare feature in Gonyleptoidea, are shared between *Hutamaia* spp. [36] and *Giupponia*. However, although these characters are compelling to phylogenetically relate *Giupponia* to Ampycidae, on the other hand, the former lacks the typical spiniform apophysis on the anal operculum.

Finally, Tricommatinae is a taxonomically convoluted subfamily that remained obscure until almost a decade ago, when [26] proposed a new circumscription based on the characterization of its type species. Tricommatinae also presents many penial features pointed out in Ampycidae, but the ventral plate is pear-shaped, and the MSA–B are not displaced from the basal lobes. Tricommatinae also exhibit an undivided scutal area I and wide scutal grooves (e.g., *Voriax popeye* Kury, 2014) with discrete sexual dimorphism on leg IV. Should *Giupponia* be a Tricommatinae, it would be relatively large compared to most members of the subfamily, but its geographical distribution would be congruent [26].

Considering the currently available data, the male genital morphology of *Giupponia* suggests possible affinities either with Ampycidae or Tricommatinae. However, its remarkable troglomorphic features may obscure phylogenetically informative characters and hinder a confident placement based solely on morphology. Biogeographically, *Giupponia* occurs in the transitional Caatinga (Brazilian tropical dry forest) to Cerrado (Brazilian savanna), close to the northeastern Atlantic Forest of Bahia, a region that coincides with the distribution of Tricommatinae, whereas Ampycidae are predominantly Andean–Amazonian. This pattern of distribution further supports a closer relationship with Tricommatinae. Nevertheless, given the morphological ambiguity, *Giupponia* is a strong candidate for inclusion in a comprehensive cladistic analysis using genomic or gene marker data, which may corroborate its affinities with Tricommatinae or Ampycidae, or even reveal that it represents an independent lineage within Gonyleptoidea. We hope to address this issue in future collaborations, as we gather the sequence from the needed species to perform an adequate phylogenetic analysis.

### 4.2. Troglomorphisms

The most conspicuous troglomorphisms are the reduction or complete absence of eyes and body pigmentation, as documented in a wide variety of obligate subterranean fauna, including flatworms, arachnids, insects, myriapods, crustaceans, fish, and salamanders [3,4,21,38,39,40]. Other common troglomorphic traits include the elongation of appendages such as legs, pedipalps, and antennae, as well as an increase in the number of mechano- and chemoreceptors, which are frequently observed in exclusive and obligate subterranean fauna.

Behavioral and physiological troglomorphisms have also been proposed; however, these traits are more difficult to recognize and classify because they generally require a phylogenetic framework for proper analysis [41]. In such cases, the most reliable approach is the comparison between troglobitic taxa and their closest epigean relatives. A decrease in body size has also been suggested as a troglomorphic trait in certain groups, such as snails [42,43].

Among Neotropical arachnids, troglomorphic traits such as the reduction or absence of body pigmentation and eyes, as well as the elongation of legs and pedipalps, are documented in several troglobitic taxa, including spiders (e.g., *Ctenus igatu* Polotow, Cizauskas & Brescovit, 2022; *Pinelema elinae* Brescovit, Gallão & Cizauskas, 2023), scorpions (*Troglorhopalurus translucidus* Lourenço, Baptista & Giupponi, 2004), and pseudoscorpions (*Spelaeobochica muchmorei* Andrade & Mahnert, 2003).

In Opiliones, three gonyleptid genera stand out due to their conspicuous morphological troglomorphisms: *Pachylospeleus strinatii* Šilhavý, 1974, *Iandumoema* Pinto-da-Rocha, 1997 and *Giupponia*. All of those exhibit elongation of appendages. Species of *Iandumoema* exhibit a marked reduction in eyes size, whereas species of *Giupponia* show a complete absence of eyes, in addition to a lack of body pigmentation [5,10,17,18].

Identifying troglomorphisms can be challenging, particularly in the case of behavioral and physiological traits; however, even morphological characters may be difficult to observe and classify. In Opiliones, the ocularium is a raised structure located between the eyes and may be unarmed or bear one central or two paramedian armatures. In troglobitic species, the armature of the ocularium may be considerably longer than in epigean relatives, as demonstrated for *Iandumoema* [7], and it is interpreted as a specialization within the group, shared homoplastically with *Guaraniticus lesserti* Mello-Leitão, 1933 (an epigean gonyleptid). This condition is also shared with the troglobitic *Giupponia*. The long, central armature in those harvestmen possibly would increase the area to harbor more sensorial receptors, such as chemical and mechanical ones, as seen in the elongation of the appendages, mainly the legs. Although this character has not yet been tested in a phylogenetic context, it is plausible to interpret the enlarged apophysis on ocularium in *Giupponia* as a troglomorphic trait, in the same fashion as *Iandumoema*.

It is noteworthy that a contrasting pattern of the ocularium armature occurs in *P. strinatii*, lacking armature entirely. Curiously, the phylogenetically closest species to it, *Hypophyllonomus longipes* Giltay, 1928 [29], bears a central armature in the ocularium. In Stygnopsidae, the basalmost monophyletic Gonyleptoidea family [44], we can see a similar situation. Many epigean stygnopsids bear a central armature on the ocularium that is quite developed in troglomorphic species, such as *Tonalteca* [44] and *Serrobunus* [45], but others, such as *Troglostygnopsis* [46], the ocularium is completely unarmed. It would be interesting to study the forces that drive the development of the ocularium’s armature or its reduction, and the first step would be addressing the phylogenetic placement of the troglomorphic species for meaningful comparisons. This is the case of *Giupponia*, as its epigean relatives remain an unsettled issue, hampering further inferences.

## 5. Conclusions

With the description of *G. lemmyi* sp. nov. and *G. ozzyi* sp. nov., the genus *Giupponia*, which had remained monotypic since its original description more than two decades ago, now comprises three species. All known species occur in caves within the Serra do Ramalho karst area, northeastern Brazil. This region represents one of the most significant hotspots of subterranean fauna in the country, harboring numerous troglobitic and troglophilic species. However, despite its high biological relevance, the caves in this area currently lack formal legal protection. This situation underscores the urgent need for effective conservation measures and highlights the critical importance of safeguarding this unique subterranean biodiversity.

## Figures and Tables

**Table 1:** Measurements of the body of males of *Giupponia lemmyi* sp. nov. (N = 2) in millimeters. Holotype measurements are in parentheses.

CL	CW	DSL	DSW
1.12–(1.18)	(1.44)–1.46	2.74–(2.76)	1.85–(1.85)

**Table 2:** Measurements of the legs I–IV of males of *Giupponia lemmyi* sp. nov. (N = 2) in millimeters. Holotype measurements are in parentheses.

	Tr	Fe	Pa	Ti	Me	Ta
Leg I	0.56–(0.56)	3.82–(4.06)	0.91–(1.00)	2.75–(2.85)	5.03–(5.24)	2.59–(2.64)
Leg II	0.59–(0.60)	7.09–(7.24)	1.01–(1.09)	5.91–(6.06)	7.35–(7.69)	(8.26)–8.31
Leg III	0.62–(0.65)	4.74–(5.09)	1.03–(1.15)	2.75–(3.14)	5.00–(5.41)	2.75–(2.85)
Leg IV	0.65–(0.68)	6.62–(6.87)	1.09–(1.15)	3.79–(4.12)	6.51–(6.88)	3.75–(3.82)

**Table 3:** Measurements of the body of female paratypes of *Giupponia lemmyi* sp. nov. in millimeters.

CL	CW	DSL	DSW
1.21	1.59	3.03	2.12

**Table 4:** Measurements of the legs I–IV of the female paratype of *Giupponia lemmyi* sp. nov. in millimeters.

	Tr	Fe	Pa	Ti	Me	Ta
Leg I	0.62	4.00	1.00	2.87	5.21	2.41
Leg II	0.68	7.24	1.12	6.11	7.35	8.05
Leg III	0.69	4.81	1.07	2.97	5.18	2.71
Leg IV	0.72	6.74	1.15	4.06	6.76	3.62

**Table 5:** Measurements of the body of males of *Giupponia ozzyi* sp. nov. (N = 2) in millimeters. Holotype measurements are in parentheses.

CL	CW	DSL	DSW
(1.03)–1.11	1.44–(1.47)	(2.58)–2. 61	(1.75)–1.78

**Table 6:** Measurements of the legs I–IV of males of *Giupponia ozzyi* sp. nov. (N = 2) in millimeters. Holotype measurements are in parentheses.

	Tr	Fe	Pa	Ti	Me	Ta
Leg I	0.50–(0.53)	3.91–(3.91)	(0.94)–1.00	(2.85)–2.88	(4.89)–5.50	2.53–(2.56)
Leg II	(0.59)–0.61	6.88–(6.91)	(1.15)–1.18	5.71–(5.76)	6.94–(7.12)	7.85–(7.88)
Leg III	(0.56)–0.59	(4.76)–4.79	(1.09)–1.15	2.82–(2.94)	(4.85)–4.94	2.76–(2.88)
Leg IV	0.65–(0.68)	(6.29)–6.41	(1.15)–1.18	3.85–(3.88)	(6.21)–6.26	3.79–(3.81)

**Table 7:** Measurements of the body of the female paratype of *Giupponia ozzyi* sp. nov. in millimeters.

CL	CW	DSL	DSW
1.06	1.39	2.64	1.75

**Table 8:** Measurements of the legs I–IV of the female paratype of *Giupponia ozzyi* sp. nov. in millimeters.

	Tr	Fe	Pa	Ti	Me	Ta
Leg I	0.44	3.53	0.97	2.56	4.44	2.24
Leg II	0.53	6.24	1.06	5.09	5.94	7.18
Leg III	0.56	4.29	0.91	2.59	4.50	2.53
Leg IV	0.56	5.71	1.06	3.53	5.62	3.24

## Data Availability

The original contributions presented in this study are included in the article. Further inquiries can be directed to the corresponding author.
